# Synthesis of enantiopure sugar-decorated six-armed triptycene derivatives

**DOI:** 10.3762/bjoc.9.278

**Published:** 2013-11-08

**Authors:** Paola Bonaccorsi, Maria Luisa Di Gioia, Antonella Leggio, Lucio Minuti, Teresa Papalia, Carlo Siciliano, Andrea Temperini, Anna Barattucci

**Affiliations:** 1 Dipartimento di Scienze chimiche, Università di Messina, viale F. Stagno d’Alcontres 31, 98166 Messina, Italy; 2 Dipartimento di Farmacia e Scienze della Salute e della Nutrizione, Università della Calabria, Edificio Polifunzionale, 87030 Arcavacata di Rende, Italy; 3 Dipartimento di Chimica, Università di Perugia, via Elce di Sotto 8, 06123 Perugia, Italy; 4 Dipartimento di Scienze del Farmaco e Prodotti per la Salute, Università di Messina, villaggio SS. Annunziata, 98168 Messina, Italy; 5 Dipartimento di Chimica e Tecnologia del Farmaco, Università di Perugia, via del Liceo 1, 06123 Perugia, Italy

**Keywords:** azide derivatives, click chemistry, glycoconjugates, 2-propyn-1-yl β-D-glycopyranosides, triptycene

## Abstract

A new class of molecules with a triptycene rigid core surrounded by six monosaccharide residues was synthesized. Hexakis(bromomethyl) substituted triptycene was converted into a six-armed triptycene azide (2,3,6,7,14,15-hexakis(azidomethyl)-9,10-dihydro-9,10-[1’,2’]benzenoanthracene). The key step of the synthesis was the cycloaddition of the azide to 2-propyn-1-yl β-D-gluco- or galactopyranosides. All products were isolated in good yields and were fully characterized.

## Introduction

**Figure 1:**
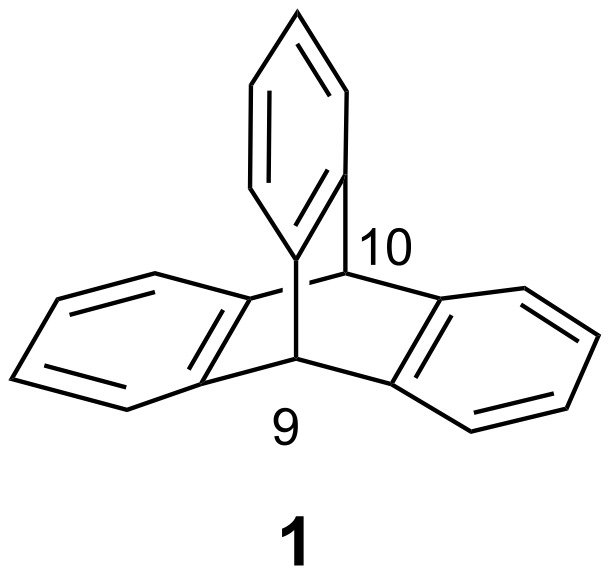
Structure of triptycene (**1**).

Triptycene (**1**), with its three arene units fused to the bicyclo[2.2.2]octa-2,5,7-triene system appears as a paddle wheel on closer inspection (Figure 1). The geometric features of its skeleton with a *D*_3_*_h_* symmetry have attracted a lot of attention and its derivatives have been used for applications in supramolecular chemistry, material chemistry, and as molecular machines [1–2]. The interest in this three-dimensional rigid platform is also due to the easy functionalization of either its arene units or the bridged positions of its bicyclooctatriene system. This allows for the development of a range of structurally different triptycene-based derivatives [1].

Triptycene derivatives have also been used as building blocks for the synthesis of new molecules in host–guest chemistry. Moreover, they have been investigated for their potential applications in molecular recognition, showing powerful complexation abilities toward different kinds of organic guests [3–4].

Many biological processes, such as cell–cell communication, immune response and cancer metastasis, are controlled by recognition phenomena between carbohydrates and proteins. Generally, individual sugar units exhibit weak binding affinities to complementary proteins, while systems that incorporate several carbohydrate units, attached to an appropriate scaffold or self-assembled in nanoparticles, lead to higher binding affinities owing to the sum of the individual interactions [5]. The synthesis of sugar-decorated molecular systems represents a significant tool in glycobiology and glycomic research fields [6]. The general prototype of a glycoconjugate comprises a core molecule that serves as an oligovalent scaffold, a number of sugar moieties, and suitable spacers which link the sugar moieties to the central core. Several examples of such molecular architectures have been obtained, and it has been demonstrated that these compounds are well-suited for the binding of lectins because of the glycoside cluster effect [7–11]. These glycoconjugates showed their potentialities not only in the interpretation of molecular recognition events but also in biotechnological, pharmaceutical and medical fields [12].

Our research interest is focused on the synthesis of new glycoconjugates and the study of their biological properties [13–14]. Accordingly, we developed a methodology for obtaining molecules showing a rigid lipophilic core represented by the triptycene skeleton that is linked, by the arene rings, to six sugar moieties through six triazole units as spacers. To the best of our knowledge, this is the first example of six-armed carbohydrate-substituted triptycenes, which appear as promising candidates for the development of new supramolecular systems with specific properties.

## Results and Discussion

**Scheme 1:**
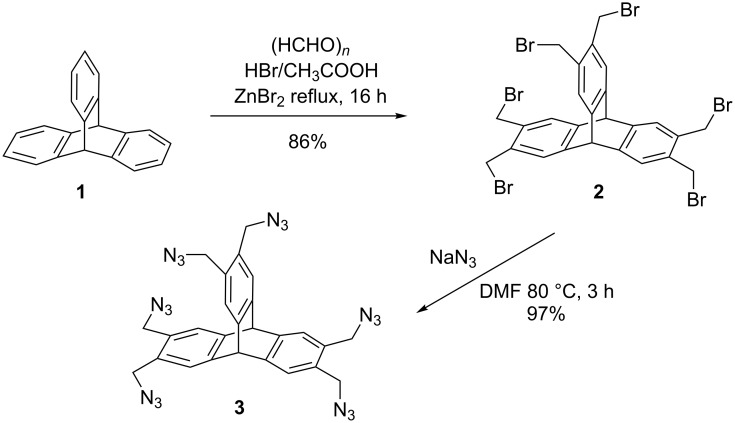
Synthesis of six-armed triptycene azide **3**.

The Huisgen 1,3-dipolar cycloaddition reaction, which represents the key step of click chemistry, can be easily applied for decorating even complex molecular systems. The reaction can be conducted in various solvents and is compatible with several functional groups. The obtained triazole ring is as stable as the most common amide bonds [15–17]. Thus, we decided to use click chemistry for the preparation of sugar-decorated triptycene derivatives. We also reasoned on the opportunity to link a significant number of carbohydrate moieties to the triptycene platform. For this reason we chose to multi-functionalize the three aromatic rings fused to the bicyclo[2.2.2]octatriene system. Triptycene (**1**) was involved in the synthetic pathway shown in Scheme 1 for the preparation of the azide derivative **3**.

For the introduction of the bromomethylene groups into the triptycene skeleton we used a literature procedure [18] that we adapted to the starting product **1**. Triptycene derivative **2,** obtained in high yield, showed a very simple ^1^H NMR spectrum that confirmed the preserved *D*_3_*_h_* symmetry and the disposition of the bromomethyl substituents depicted in Scheme 1. Bĕlohradský, Kilså, Nielsen et al. [19] have previously reported a different synthetic route to compound **2**, performed in two steps from 9,10-dihydro-9,10-[1',2']benzenoanthracene-2,3,6,7,14,15-hexacarboxylic acid through its hexaethyl ester. However, their yields were very low, 17% in the first step and 26% in the subsequent LiAlH_4_ reduction/HBr bromination. Moreover 9,10-dihydro-9,10[1',2']-benzenoanthracene-2,3,6,7,14,15-hexacarboxylic acid has to be prepared, while our procedure consists of a single step (Scheme 1), has a yield of 86%, and starts from the commercially available triptycene (**1**). The reaction of compound **2** with NaN_3_ [20] led to the formation of the not yet known hexa-azide derivative **3** in almost quantitative yield as a colorless solid easily purified by column chromatography.

**Scheme 2:**
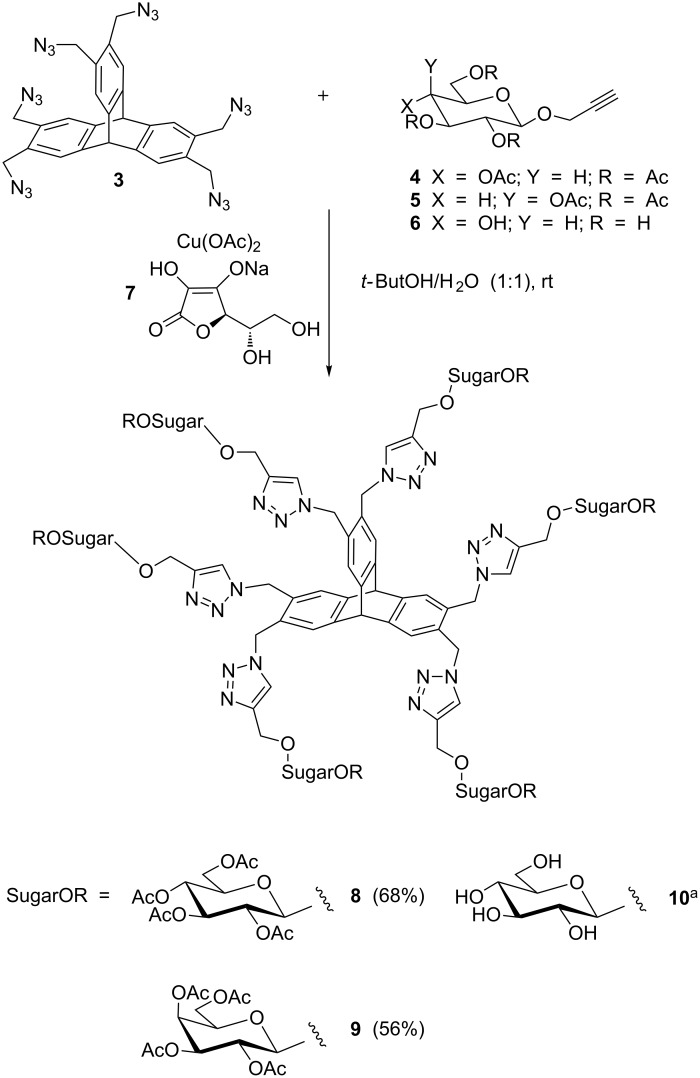
Synthesis of six-armed triptycene derivatives **8**–**10** from triptycene azide **3**. ^a^Not easily isolable in pure form (see Results and Discussion section, last paragraph).

Azide acceptors, the 2,3,4,6-tetraacetyl-2-propyn-1-yl-β-D-glycopyranosides **4** and **5** (Scheme 2), were prepared following literature procedures [21–22]. We also decided to subject compound **3** to the cycloaddition with sugar **6** that was obtained by the almost quantitative deacetylation of 2,3,4,6-tetraacetyl-2-propyn-1-yl-β-D-glucopyranoside (**4**).

Firstly, we proceeded with the setup of the click cycloaddition between triptycene hexa-azide **3** and 2,3,4,6-tetraacetyl-1-prop-2-ynyl-β-D-glucopyranoside (**4**). We attempted to perform the reaction by using ascorbic acid, Na_2_CO_3_ and CuSO_4_·5H_2_O [23], but no formation of the expected product was observed and the unreacted acceptor **4** was recovered, while the azide derivative **3** could not be detected in the crude reaction mixture. New reaction conditions were tested, in which CuSO_4_ was substituted by CuI and Na_2_CO_3_ was substituted by NEt_3_ [24], but the work-up of the reaction appeared difficult, and no final product of the click reaction was obtained. Finally, we decided to adopt the synthetic conditions shown in Scheme 2 [25–26]. The triptycene derivative **3** was reacted with the unsaturated sugar acceptor **4** in the presence of Cu(OAc)_2_ and sodium ascorbate **7** and, after 24 h of stirring at rt, the reaction appeared complete. Trace amounts of decomposed acceptor **4** were isolated. The sugar-decorated six-armed triptycene derivative **8** was easily purified and obtained in very good yield. The click cycloaddition of compound **3** with acceptor **5** was complicated by the formation of a bulky, solid aggregate which is the reason of the lower yields obtained in this step for the preparation of sugar-decorated triptycene **9** with respect to the one performed with acceptor **4**. However, even in this case, results appeared significant and compound **9** was easily isolated by column chromatography.

**Scheme 3:**
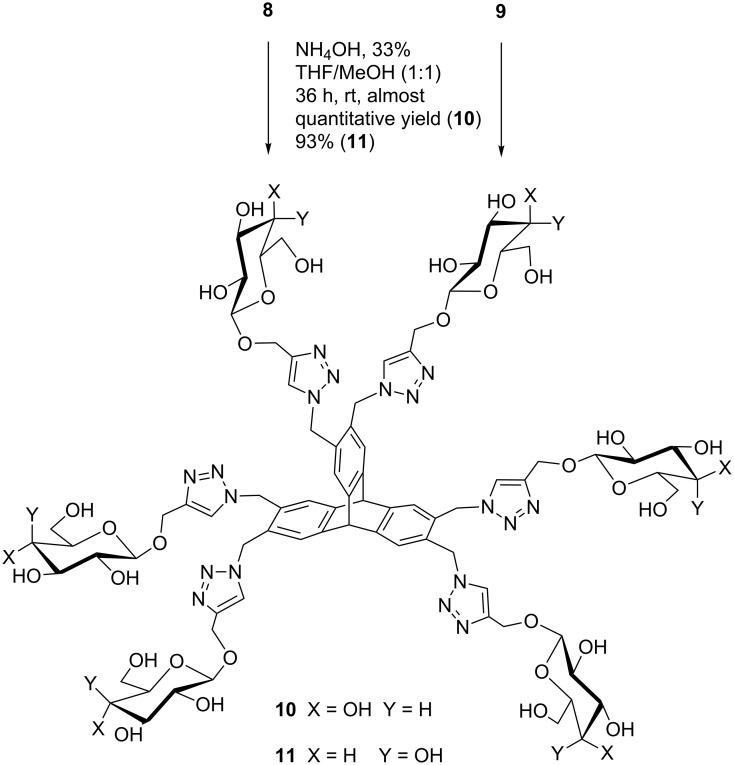
Deacetylation of target compounds **8** and **9** to **10** and **11,** respectively.

The reaction of azide **3** with sugar acceptor **6** was also performed under the conditions described above, and an inspection of the ^1^H NMR of the crude reaction mixture clearly indicated the formation of the desired triptycene derivative **10**. The unreacted compound **3** was recovered after repeated washings of the crude with DCM, but the separation of compound **10** from the catalyst appeared highly demanding. Several attempts of crystallization of compound **10** from mother liquor failed, so we decided to subject the *O*-acetylated D-glucose substituted triptycene derivative **8** to a deprotection reaction, following a literature procedure [22]. After 36 h at rt and a number of washings with MeOH to eliminate acetamide, the sugar-decorated triptycene **10** was obtained from compound **8** in almost quantitative yield. The same procedure of deprotection allowed the formation of **11** from compound **9** (Scheme 3).

## Conclusion

In conclusion, we have described an efficient approach to the synthesis of enantiopure six-armed sugar-decorated triptycene derivatives **8**–**11**. These compounds are a novel class of molecules with potential applications not only as glycoconjugates in glycochemistry, but also as interesting candidates for host–guest interactions by presenting a rigid lipophilic core surrounded by hydrophilic sugar moieties assembled on a rotor skeleton. The six-armed sugar-decorated triptycene derivatives might constitute a new tool for the study of molecular recognition.

## Experimental

**General methods.** All commercial reagents and solvents (AR, LabScan Ltd.; SpS, Romil Ltd.) were used without further purification. All syntheses were carried out under atmospheric conditions unless otherwise noted. Analytical TLC was performed on Aldrich silica gel 60 F_254_ plates. Products were visualized by UV or vanillin [1 g dissolved in MeOH (60 mL) and conc. H_2_SO_4_ (0.6 mL)]. Column chromatography was performed on Aldrich 60 silica gel (40–63 μm). ^1^H and ^13^C NMR measurements were performed in CDCl_3_ or DMSO-*d*_6_ solutions at 300.1 and 75.5 MHz or at 500.1 and 125.7 MHz, respectively. All chemical shifts are reported in parts per million (δ/ppm), downfield to tetramethylsilane (Me_4_Si) as an internal standard (δ = 0.00 ppm), or referenced to the residual solvent CDCl_3_ (^1^H NMR 7.27 ppm and ^13^C NMR 77.0 ppm) and DMSO-*d*_6_ (^1^H NMR 2.50 ppm and ^13^C NMR 39.5 ppm). NMR peak assignments were performed through APT (Attached Proton Test) experiments, homonuclear (COSY, COrrelationSpectroscopY) and heteronuclear correlation (^1^H–^13^C) spectra. Melting points were determined on a Kofler hot-stage apparatus and are uncorrected. Optical rotations were measured at rt (1 dm cell length).

**2,3,6,7,14,15-Hexakis(bromomethyl)-9,10-dihydro-9,10-[1’,2’]benzenoanthracene** (**2**). Triptycene (**1**) (0.90 g, 3.54 mmol) and paraformaldehyde (2.34 g, 77.92 mmol of monomer) were dissolved in HBr solution in AcOH (12.5 mL ≥33%, 71.38 mmol). ZnBr_2_ (2.63 g, 11.68 mmol) was slowly added to the solution, under stirring, at rt. The mixture was heated under reflux at 105 °C for 16 h, and the reaction was monitored by TLC (hexane/DCM 60:40). The final mixture was dissolved in DCM (25 mL), washed with a saturated solution of NaHCO_3_ (2 × 20 mL), dried over Na_2_SO_4_, and the solvent was removed in vacuo. The residue was purified by column chromatography (hexane with 10% DCM up to 70%) to give compound **2** (2.48 g, 3.05 mmol, 86%). *R*_f_ 0.53 (hexane/EtOAc 80:20); ^1^H NMR (CDCl_3_) δ 7.38 (s, 6H, ArH), 5.39 (s, 2H, H-9,10), 4.57 (s, 12H, 6 × CH_2_); ^13^C NMR (CDCl_3_) δ 145.0 (C-4a,4b,8a,8b,11,12), 134.0 (C-2,3,6,7,14,15), 126.5 (C-1,4,5,8,13,16), 52.5 (C-9,10), 29.6 (CH_2_). The spectra are in full agreement with published data [19].

**2,3,6,7,14,15-Hexakis(azidomethyl)-9,10-dihydro-9,10-[1’,2’]benzenoanthracene** (**3**). NaN_3_ (0.48 g, 7.38 mmol) was added to a solution of compound **2** (0.50 g, 0.62 mmol) in DMF (10 mL). The reaction mixture was heated under reflux at 80 °C for 3 h, quenched with H_2_O (2 mL), and extracted with EtOAc (3 × 10 mL). The organic layer was washed with a saturated solution of NaCl, dried over Na_2_SO_4_, and the solvent was removed in vacuo. The residue was purified by column chromatography on silica gel (hexane/EtOAc 90:10) to give compound **3** (0.35 g, 0.60 mmol, 97%) as a colorless solid. *R*_f_ 0.76 (hexane/EtOAc 80:20); ^1^H NMR (CDCl_3_) δ 7.43 (s, 6H, ArH), 5.51 (s, 2H, H-9,10), 4.37 (s, 12H, 6 × CH_2_); ^13^C NMR (CDCl_3_) δ 145.00 (C-4a,4b,8a,8b,11,12), 131.4 (C-2,3,6,7,14,15), 125.3 (C-1,4,5,8,13,16), 52.9 (C-9,10), 52.1 (CH_2_); Anal. calcd for C_26_H_20_N_18_, C, 53.42; H, 3.45; found; C, 53.09; H, 3.58.

**General procedure for the azide–alkyne cycloaddition.** Azide **3** (0.10 g, 0.17 mmol), the 2-propyn-1-yl β-D-glycopyranoside (0.84 mmol), copper(II) acetate (15 mg, 0.08 mmol) and sodium L-ascorbate (33 mg, 0.17 mmol) were suspended in distilled water/degassed *t*-BuOH (50:50, 7 mL). The reaction mixture was stirred for 24 h at rt, during which time the color of the solution changed from yellow to light green, and monitored by TLC (hexane/EtOAc 70:30). The solvents were removed in vacuo, and the residue was purified by column chromatography on silica gel (EtOAc/MeOH 95:5).

**Compound 8.** Following the general procedure, compound **8** was prepared from azide **3** and 2,3,4,6-tetraacetatyl-1-(prop-2-ynyl)-β-D-glucopyranoside (**4**), and isolated as a colorless solid (0.34 g, 0.12 mmol, 68%); mp 110–120 °C; *R*_f_ 0.34 (EtOAc/MeOH 95:5); [α]_D_ −32.1 (*c* 0.01, CHCl_3_); ^1^H NMR (CDCl_3_) δ 7.55 and 7.24 (two s, 12H, ArH and triazoleH), 5.55 (m, 12H, 6 × NCH_2_), 5.40 (s, 2H, H-9,10), 5.21 (t, *J* = 9.3 Hz, 6H, 6 × sugarH-3), 5.09 (t, *J* = 9.8 Hz, 6H, 6 × sugarH-4), 4.96 (t, *J* = 8.8 Hz, 6H, 6 × sugarH-2, 6 × sugarH-2-4), 4.83 (m, 12H, 6 × triazole linked CH_2_O), 4.69 (d, *J* = 7.3 Hz, 6H, 6 × sugarH-1), 4.25 and 4.17 (split AB system, *J*_6A,6B_ = 12.2 Hz, *J*_5,6A_ = 4.4 Hz, *J*_5,6B_ = <1.0 Hz, 12H, 6 × sugarH_2_-6), 3.75 (m, 6H, 6 × sugarH-5), 2.06, 2.04, 2.00, and 1.89 (four s, 72H, 24 × CH_3_); ^13^C NMR (CDCl_3_) δ 170.6, 170.1, 169.4, and 169.3 (CO), 145.2, (C-4a,4b,8a,8b,11,12 and triazoleqC), 130.8 (C-2,3,6,7,14,15), 125.8 (C-1,4,5,8,13,16 and triazoleCH), 100.1 (sugarC-1), 72.7 (sugarC-3), 71.9 (sugarC-5), 71.2 (sugarC-2), 68.3 (sugarC-4), 62.9 (triazole linked CH_2_O), 61.7 (sugarC-6), 50.7 and 50.3 (C-9,10 and NCH_2_), 20.5 (CH_3_); Anal. calcd for C_128_H_152_N_18_O_60_, C, 52.96; H, 5.28; found, C, 52.82; H, 5.30.

**Compound 9.** Following the general procedure, compound **9** was prepared from azide **3** and 2,3,4,6-tetraacetatyl-1-(prop-2-ynyl)-β-D-galactopyranoside (**5**), and isolated as a colorless solid (0.28 g, 0.10 mmol, 56%); mp 120–135 °C; *R*_f_ 0.34 (EtOAc/MeOH 95:5); [α]_D_ −34.7 (*c* 0.01, CHCl_3_); ^1^H NMR (CDCl_3_) δ 7.52 and 7.23 (two s, 12H, ArH and triazoleH), 5.60 (m, 12H, 6 × NCH_2_), 5.41 (m, 8H, H-9,10 and 6 × sugarH-4), 5.17 (dd, *J* = 10.2 and 7.8 Hz, 6H, 6 × sugarH-2), 5.05 (dd, *J* = 10.3 and 3.4 Hz, 6H, 6 × sugarH-3), 4.84 (m, 12H, 6 × triazole linked CH_2_O), 4.65 (d, *J* = 7.8 Hz, 6H, 6 × sugarH-1), 4.14 (m, 12H, 6 × sugarH_2_-6), 3.96 (m, 6H, 6 × sugarH-5), 2.16, 2.05, 2.00, and 1.90 (four s, 72H, 24 × CH_3_); ^13^C NMR (CDCl_3_) δ 170.4, 170.2, 170.1, and 169.5 (CO), 145.2, (C-4a,4b,8a,8b,11,12 and triazoleqC), 130.7 (C-2,3,6,7,14,15), 125.8 (C-1,4,5,8,13,16 and triazoleCH), 100.5 (sugarC-1), 70.9 and 70.7 (sugarC-3,5), 68.8 (sugarC-2), 67.0 (sugarC-4), 62.8 (triazole linked CH_2_O), 61.2 (sugarC-6), 50.8 (C-9,10 and NCH_2_), 20.6 (CH_3_); Anal. calcd for C_128_H_152_N_18_O_60_, C, 52.96; H, 5.28; found: C, 52.82; H, 5.30.

**General procedure for the deprotection of triptycene derivatives 8 and 9.** Ammonium hydroxide (15 mL, 38% in H_2_O) was added to the solution of compound **8** or **9** (0.26 g, 0.09 mmol) in THF/MeOH (50:50, 40 mL). The reaction mixture was stirred for 36 h at rt and monitored by TLC (EtOAc/MeOH 95:5). The solvents were removed in vacuo, and the colorless solid was washed with MeOH (4 × 20 mL) and centrifugated four times to remove acetamide.

**Compound 10.** Following the general procedure, compound **10** was obtained from triptycene derivative **8** and isolated as a colorless solid (0.17 g, 0.09 mmol, quantitative yield); mp 120–130 °C; [α]_D_ −25.3 (*c* 5.7 × 10^−3^, H_2_O); ^1^H NMR (DMSO-*d*_6_) δ 8.12 and 7.19 (two s, 12H, ArH and triazoleH), 5.75 (s, 2H, H-9,10), 5.65 (m, 12H, 6 × NCH_2_), 5.04–4.57 (m, 36 H, 6 × four sugarOH and 6 × triazole linked CH_2_O), 4.25 (d, *J* = 7.8 Hz, 6H, sugarH-1), 3.72–3.40 (m, 12H, 6 × sugarH_2_-6), 3.20–2.95 (m, 24H, 6 × sugarH-2-5); ^13^C NMR (DMSO-*d*_6_) δ 144.9, and 144.0 (C-4a,4b,8a,8b,11,12 and triazoleqC), 131.2 (C-2,3,6,7,14,15), 124.7 and 124.5 (C-1,4,5,8,13,16 and triazoleCH), 102.0 (sugarC-1), 76.9, 76.6, 73.3, and 70.0 (sugarC-2-5), 61.3 (triazole linked CH_2_O), 61.1 (sugarC-6), 51.0 (C-9,10), 49.5 (NCH_2_); Anal. calcd for C_80_H_104_N_18_O_36_, C, 50.74; H, 5.54; found, C, 50.62; H, 5.40.

**Compound 11.** Following the general procedure, compound **11** was obtained from triptycene derivative **9** and isolated as a colorless solid (0.16 g, 0.08 mmol, 93%); mp 180–195 °C; [α]_D_ −10.2 (*c* 3.15 × 10^−3^, H_2_O); ^1^H NMR (DMSO-*d*_6_) δ 8.11 and 7.19 (two s, 12H, ArH and triazoleH), 5.75 (s, 2H, H-9,10), 5.65 (m, 12H, 6 × NCH_2_), 4.90–4.40 (m, 36 H, 6 × four sugarOH and 6 × triazole linked CH_2_O), 4.20 (d, *J* = 6.4 Hz, 6H, sugarH-1), 3.64–3.29 (m, 36H, 6 × sugarH-2-6); ^13^C NMR (DMSO-*d*_6_) δ 144.9 and 144.1 (C-4a,4b,8a,8b,11,12 and triazoleqC), 131.1 (C-2,3,6,7,14,15), 124.6 (C-1,4,5,8,13,16 and triazoleCH), 102.7 (sugarC-1), 75.2, 73.3, 70.4, and 68.1 (sugarC-2-5), 61.3 (triazole linked CH_2_O), 60.5 (sugarC-6), 50.9 (C-9,10), 49.5 (NCH_2_); Anal. calcd for C_80_H_104_N_18_O_36_, C, 50.74; H, 5.54; found, C, 50.72; H, 5.60.

## Supporting Information

File 1 Copies of ^1^H and ^13^C NMR spectra of new compounds **3**, **8–11**.
