# Supplementary material for: Synthesis of enantiopure sugar-decorated six-armed triptycene derivatives
Source: Beilstein J Org Chem. 2013 Nov 8;9:2410–6. doi: 10.3762/bjoc.9.278 (PMC3869296; doi:10.3762/bjoc.9.278)
Supplement: File 1 — Copies of 1H and 13C NMR spectra of new compounds 3, 8–11. [file Beilstein_J_Org_Chem-09-2410-s001.pdf]

Supporting Information

for

Synthesis of enantiopure sugar-decorated six-armed triptycene derivatives

Paola Bonaccorsi*[1], Maria Luisa Di Gioia[2], Antonella Leggio[2], Lucio Minuti[3], Teresa Papalia[4], Carlo Siciliano[2], Andrea Temperini[5] and Anna Barattucci[1]

Address: [1]Dipartimento di Scienze chimiche, Università di Messina, viale F. Stagno d'Alcontres 31, 98166 Messina, Italy, [2]Dipartimento di Farmacia e Scienze della Salute e della Nutrizione, Università della Calabria, Edificio Polifunzionale, 87030 Arcavacata di Rende, Italy, [3]Dipartimento di Chimica, Università di Perugia, via Elce di Sotto 8, 06123 Perugia, Italy, [4]Dipartimento di Scienze del Farmaco e Prodotti per la Salute, Università di Messina, villaggio SS. Annunziata, 98168 Messina, Italy and [5]Dipartimento di Chimica e Tecnologia del Farmaco, Università di Perugia, via del Liceo 1, 06123 Perugia, Italy.

Email: Paola Bonaccorsi* - *pbonaccorsi@unime.it*

* Corresponding author

Copies of ^{1}H and ^{13}C NMR spectra of new compounds 3, 8–11

Table of Content

^{1}H NMR spectrum of compound **3**……………………………………………………….…………S3

^{13}C NMR spectrum of compound **3**………………………...……………..……………………..S4

^{1}H NMR spectrum of compound **8**……………………………………………..……………..S5

^{13}C NMR spectrum of compound **8**…………………………………………………...……….S6

^{1}H NMR spectrum of compound **9**………………..…………………………………..…………S7

^{13}C NMR spectrum of compound **9**……………..…………………………………………………..S8

^{1}H NMR spectrum of compound **10**………..………………………………………………………S9

^{13}C NMR spectrum of compound **10** …………………………………………...…………S10

^{1}H NMR spectrum of compound **11**……………………………………………………….....S11

^{13}C NMR spectrum of compound **11**……………………………………………………….…..S12

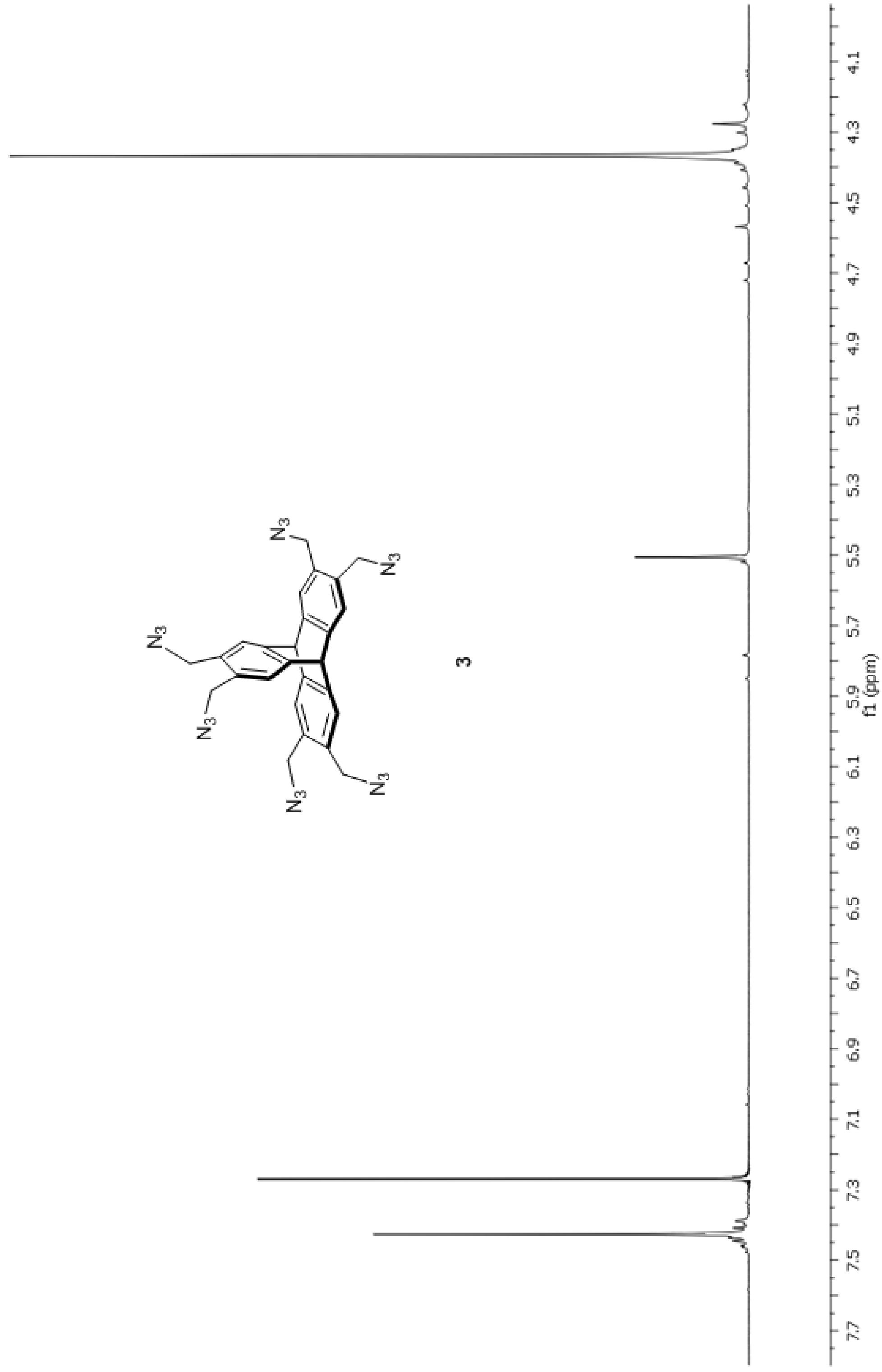

^{1}H NMR of 2,3,6,7,14,15-hexakis(azidomethyl)-9,10-dihydro-9,10-[1’,2’]benzenoantracene (**3**)

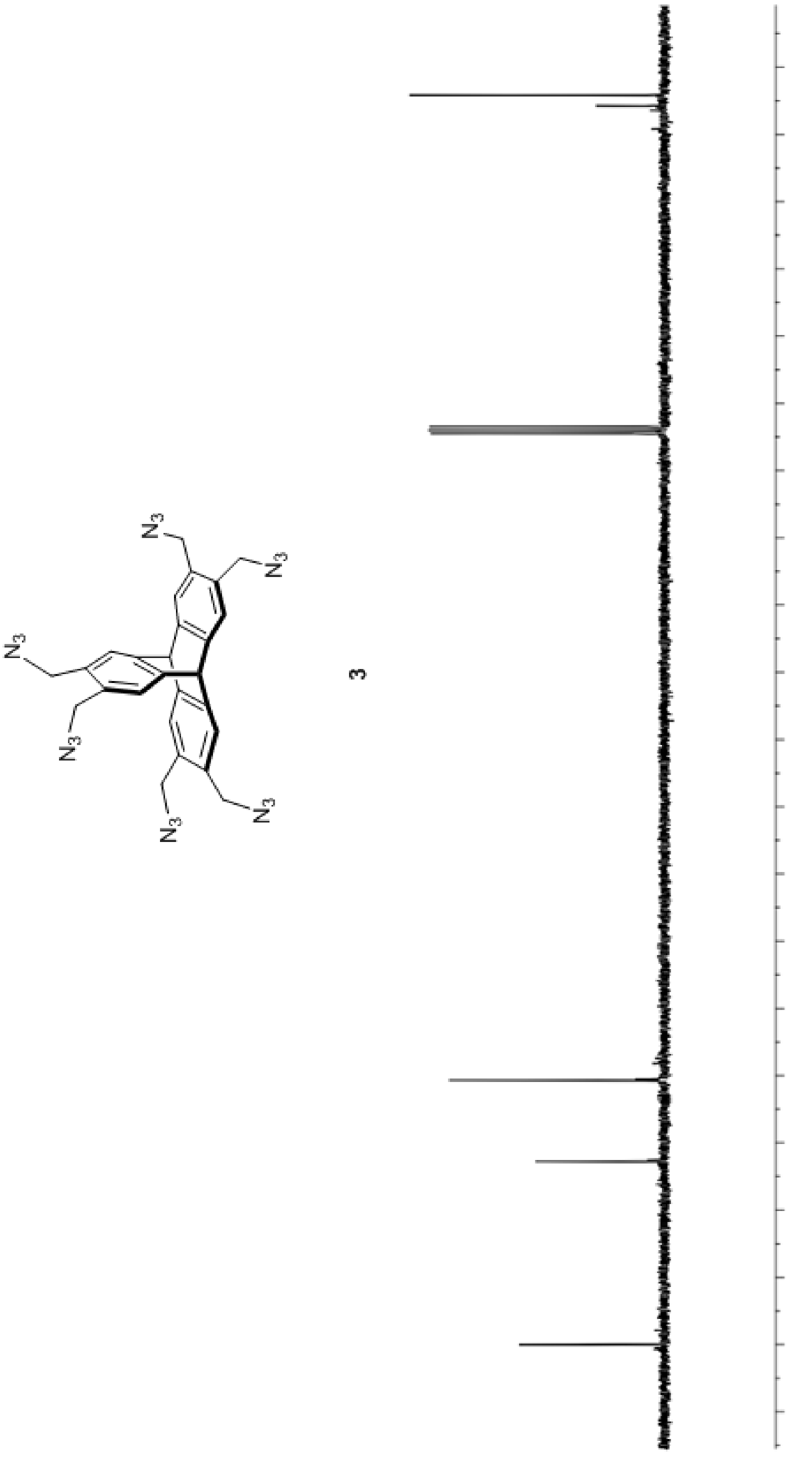

^{13}C NMR of 2,3,6,7,14,15-hexakis(azidomethyl)-9,10-dihydro-9,10-[1',2']benzenoantracene (**3**)

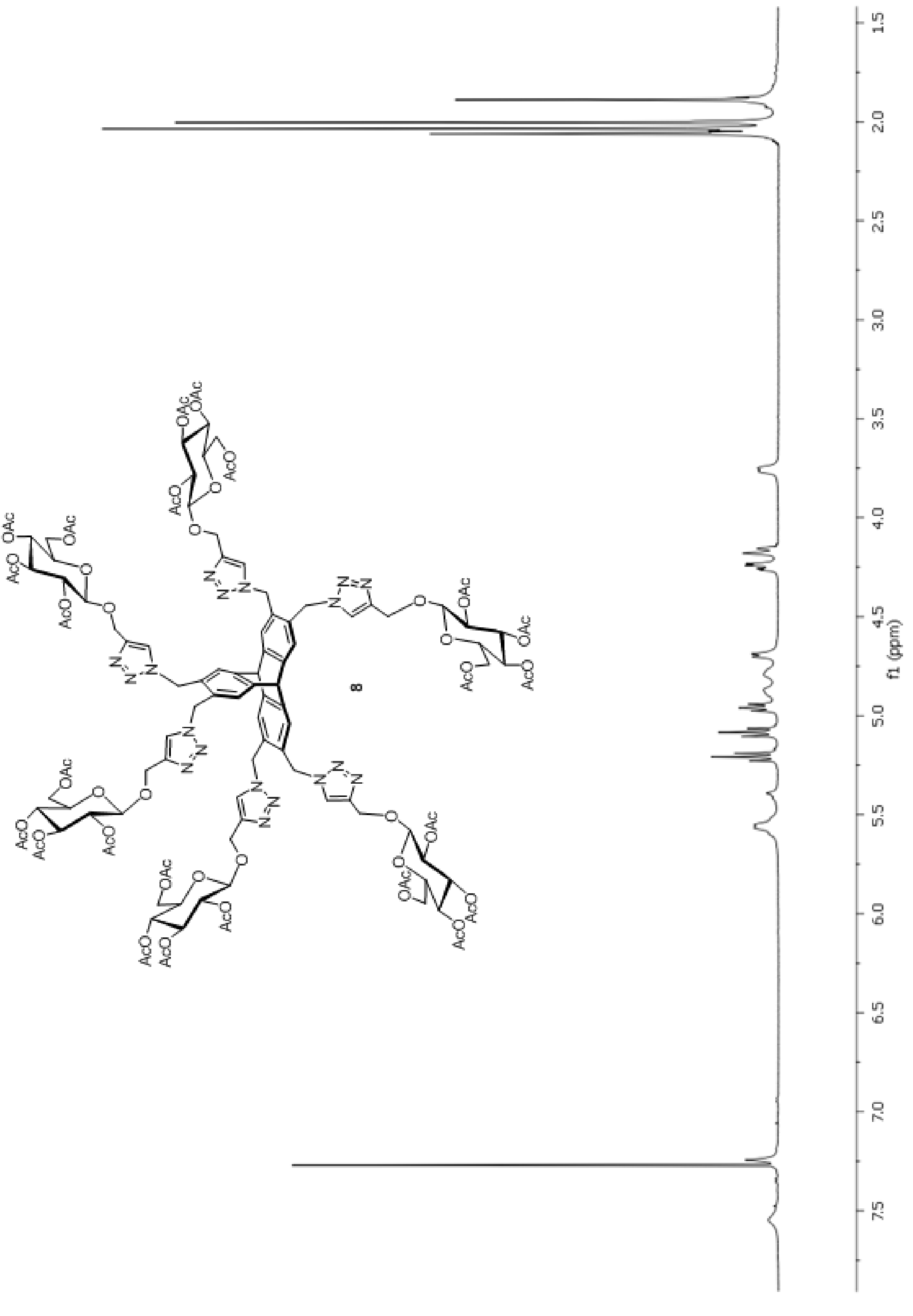

^{1}H NMR of compound **8**

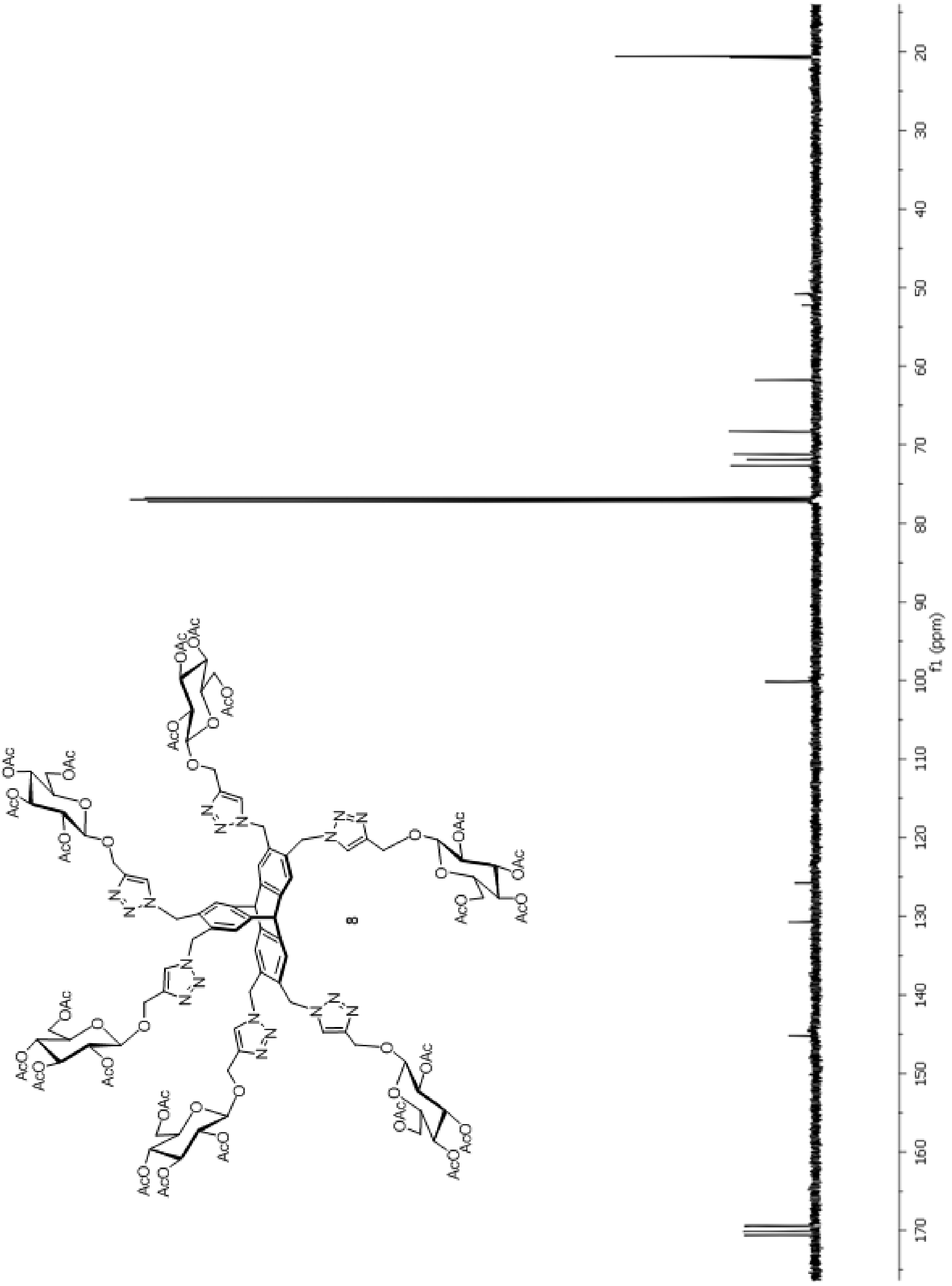

^{13}C NMR of compound **8**

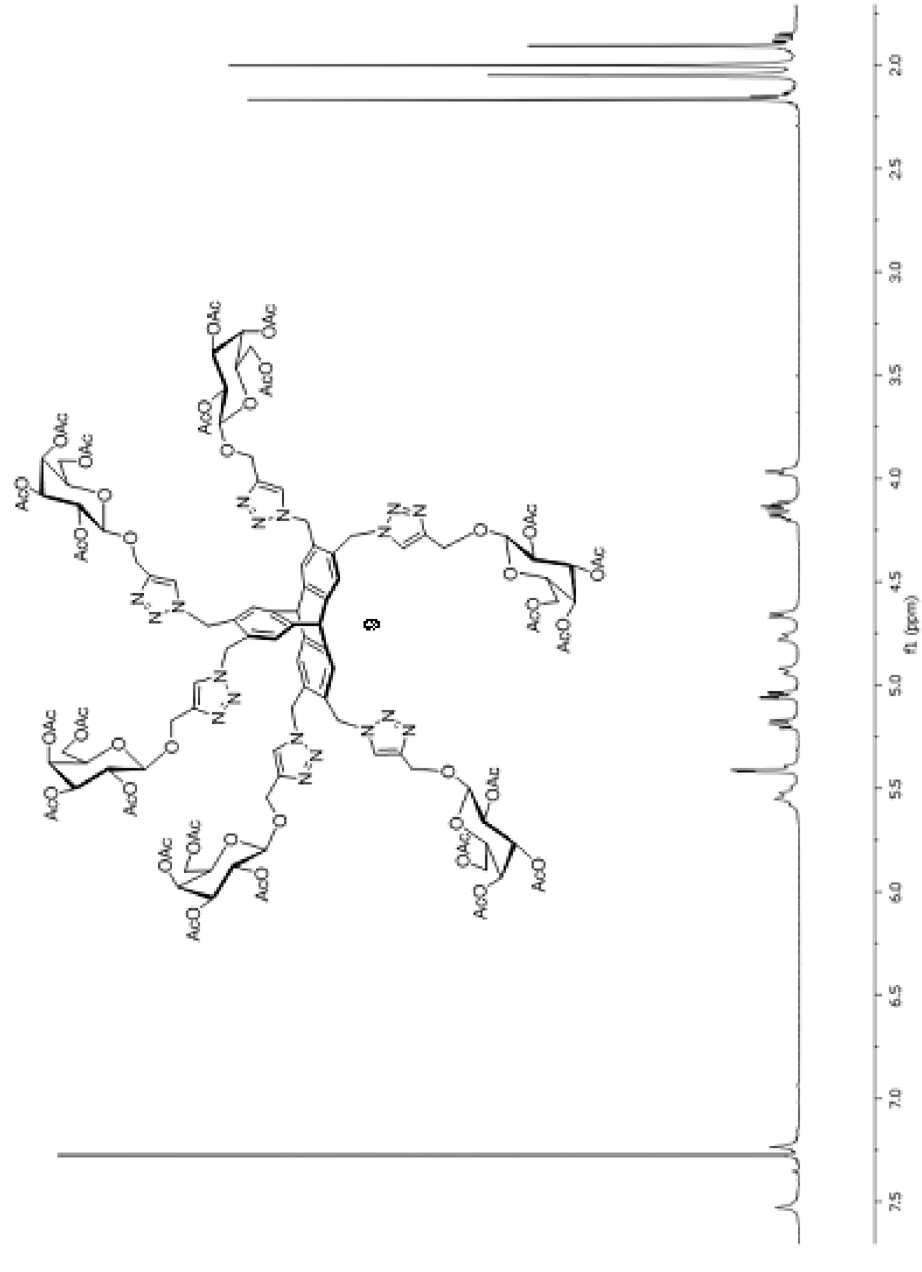

^{1}H NMR of compound **9**

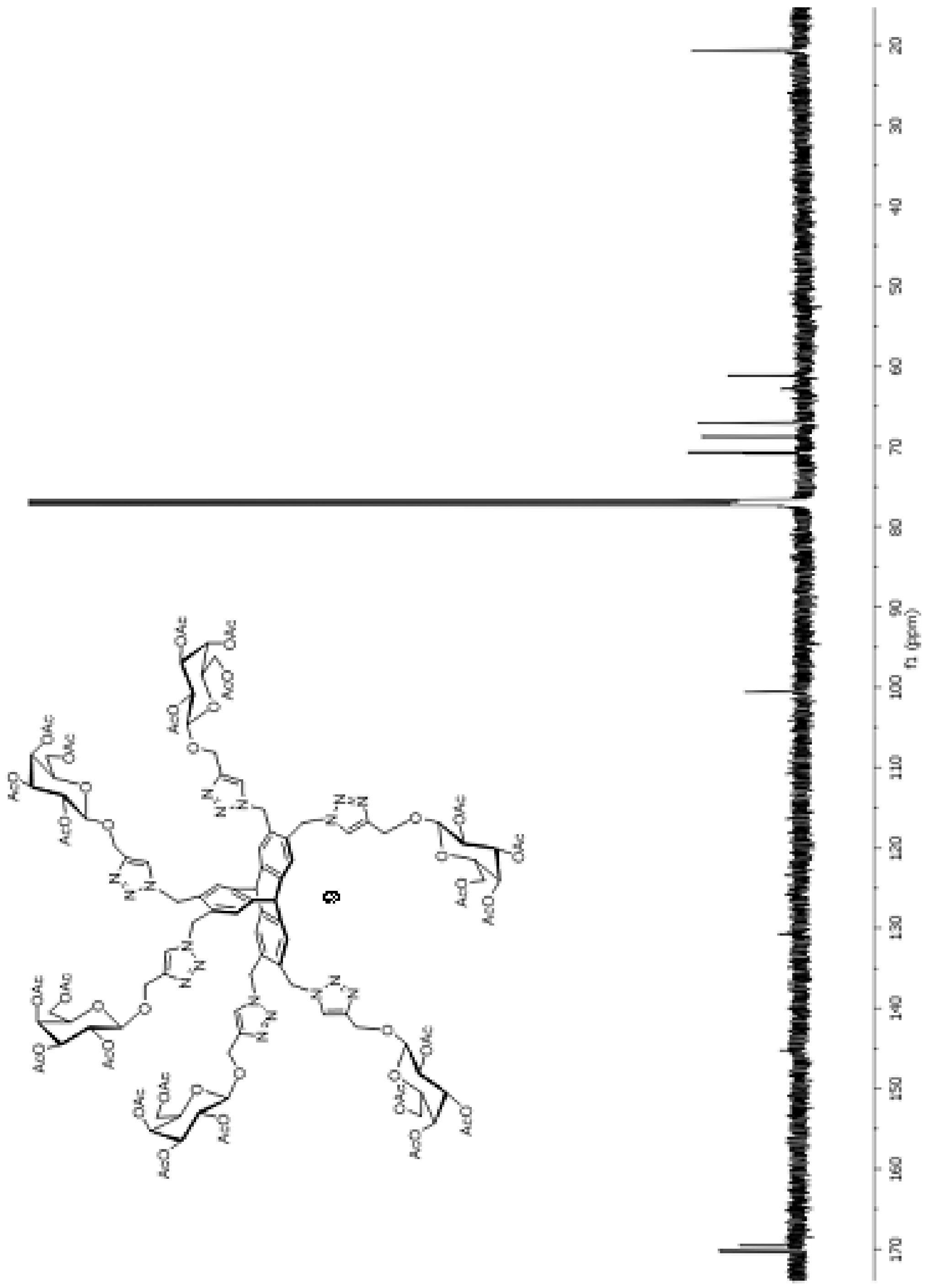

^{13}C NMR of compound **9**

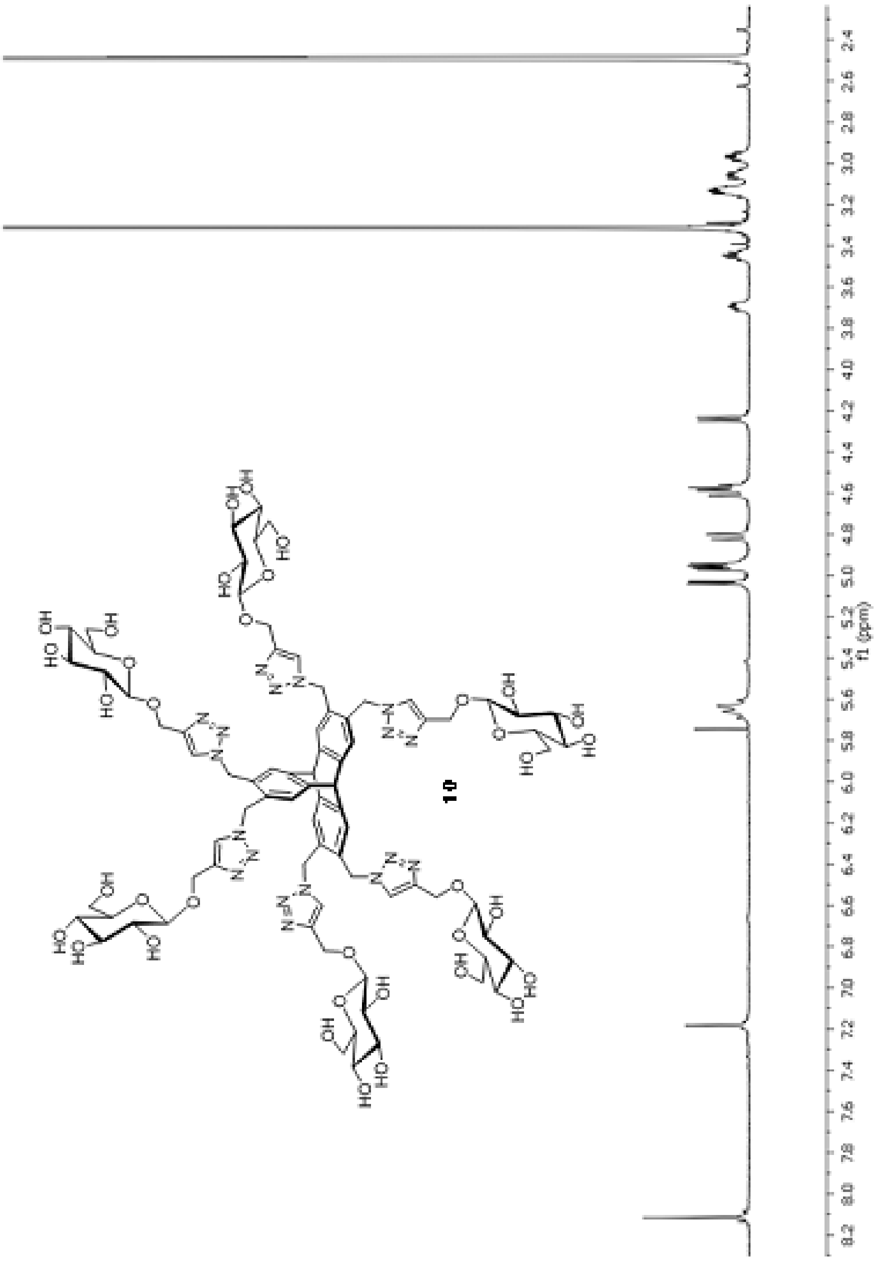

^{1}H NMR of compound **10**

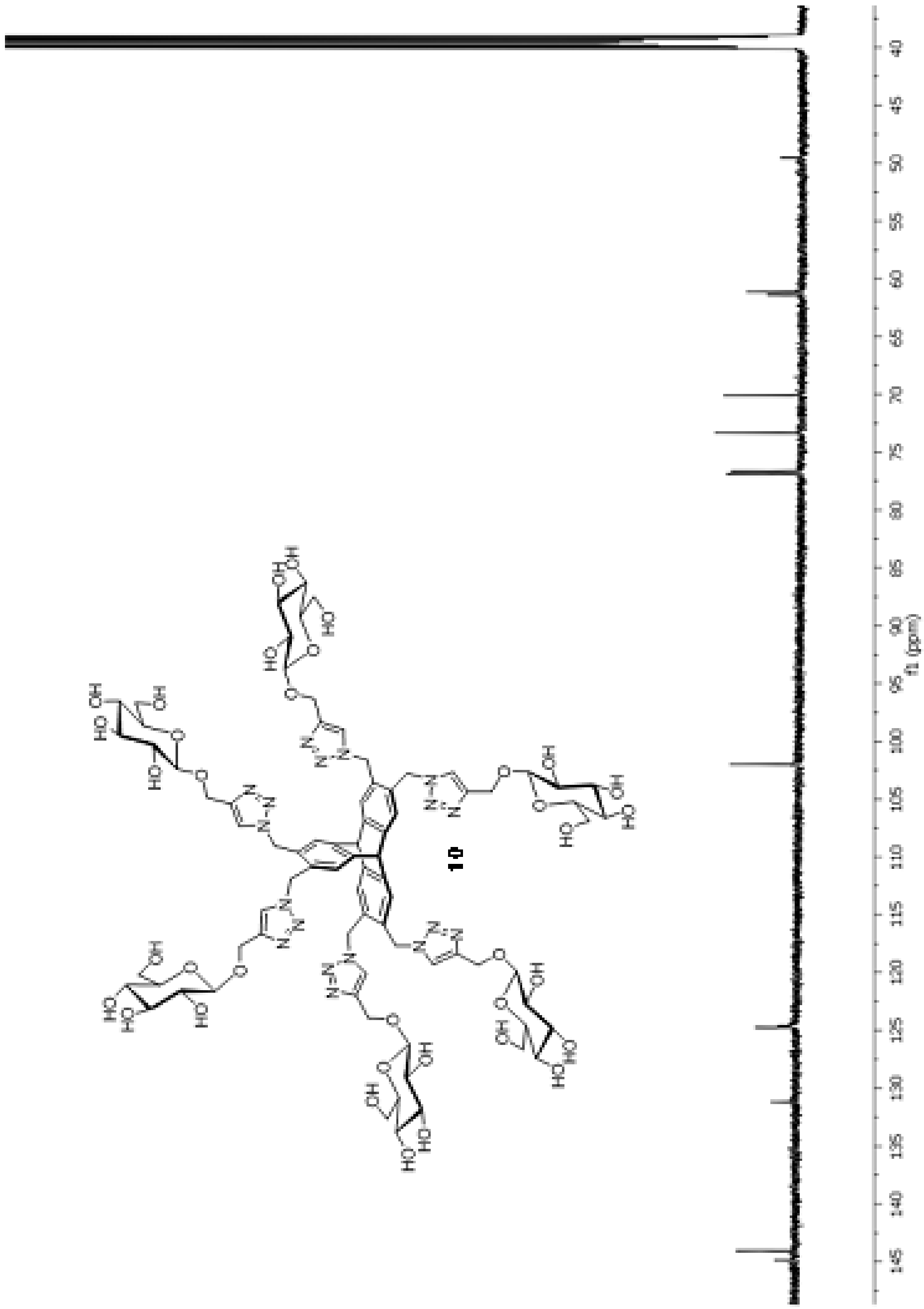

^{13}C NMR of compound **10**

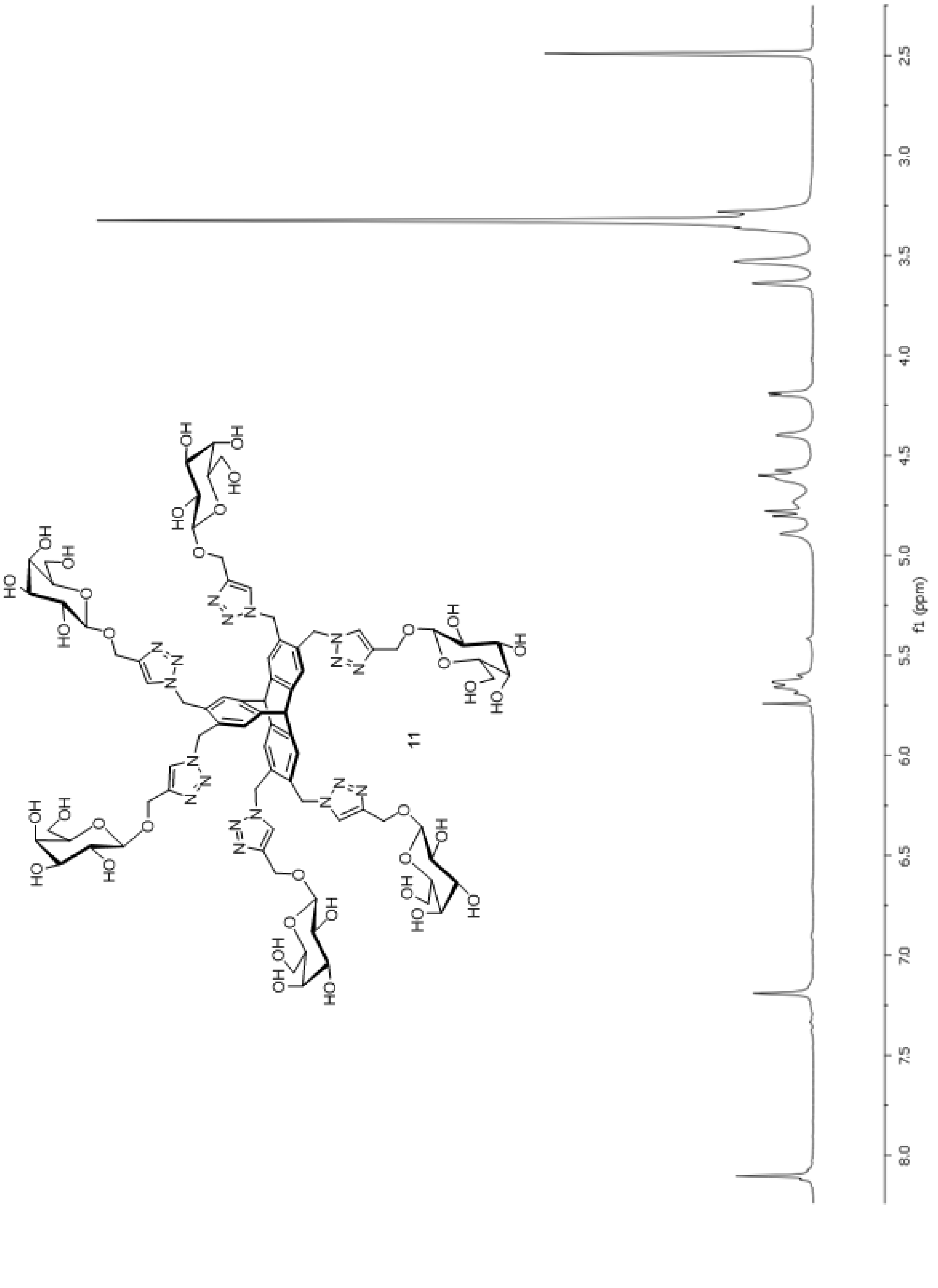

^{1}H NMR of compound **11**

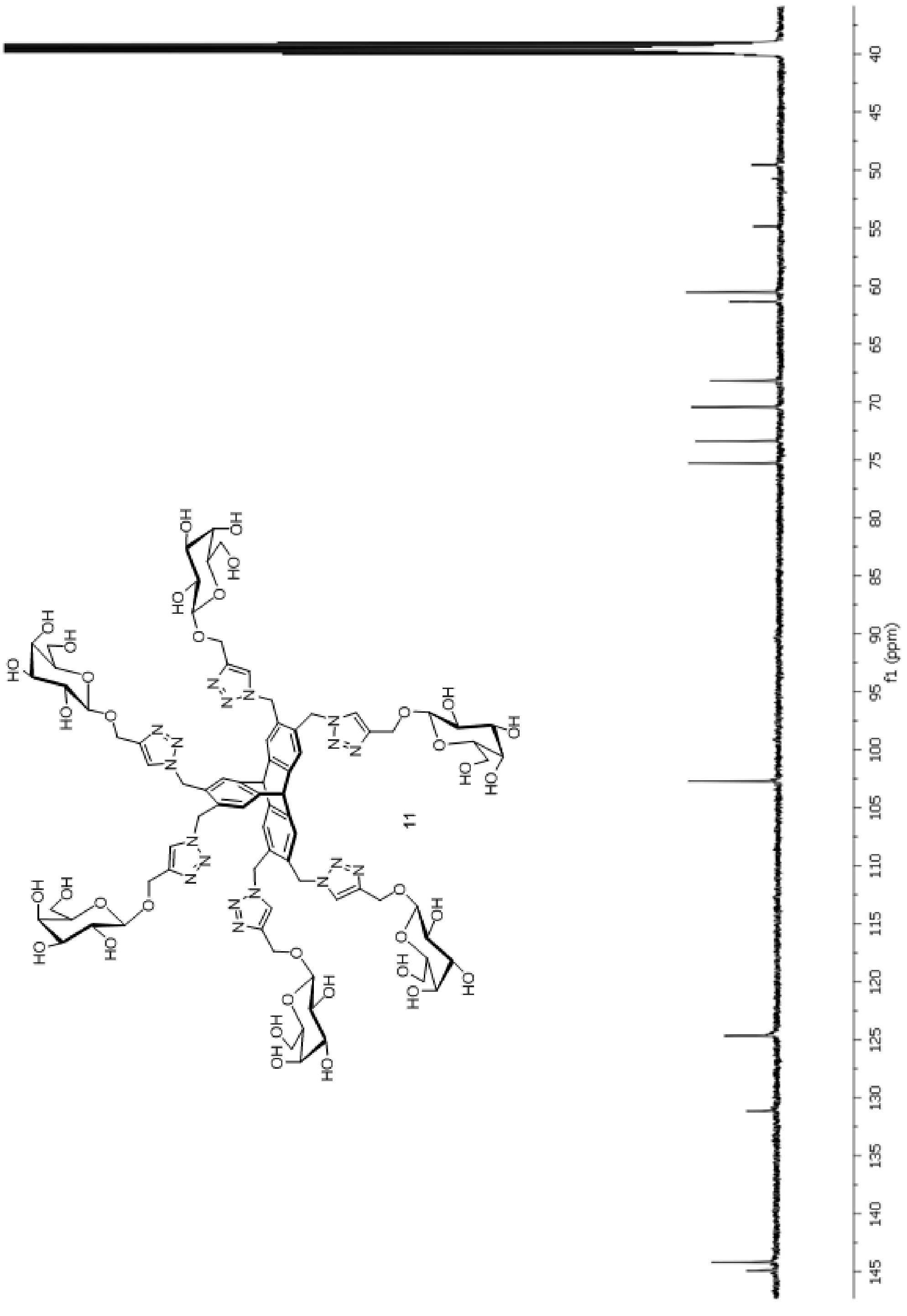

^{13}C NMR of compound **11**
